# Gender-Based Analysis of Risk Factors for Dementia Using Senior Cohort

**DOI:** 10.3390/ijerph17197274

**Published:** 2020-10-05

**Authors:** Jaekue Choi, Lee-Nam Kwon, Heuiseok Lim, Hong-Woo Chun

**Affiliations:** 1 Convergence Research Center for Diagnosis, Treatment and Care System of Dementia, Korea Institute of Science and Technology, Seoul 02792, Korea; 1diversity2@kist.re.kr (J.C.); ynkwon@kist.re.kr (L.-N.K.); 2 Future Information Research Center, Korea Institute of Science and Technology Information, Seoul 02456, Korea; 3 Department of Computer Science and Engineering, Korea University, Seoul 02855, Korea; limhseok@korea.ac.kr

**Keywords:** dementia, dementia risk factor, machine learning, deep learning, senior cohort

## Abstract

Globally, one of the biggest problems with the increase in the elderly population is dementia. However, dementia still has no fundamental cure. Therefore, it is important to predict and prevent dementia early. For early prediction of dementia, it is crucial to find dementia risk factors that increase a person’s risk of developing dementia. In this paper, the subject of dementia risk factor analysis and discovery studies were limited to gender, because it is assumed that the difference in the prevalence of dementia in men and women will lead to differences in the risk factors for dementia among men and women. This study analyzed the Korean National Health Information System—Senior Cohort using machine-learning techniques. By using the machine-learning technique, it was possible to reveal a very small causal relationship between data that are ignored using existing statistical techniques. By using the senior cohort, it was possible to analyze 6000 data that matched the experimental conditions out of 558,147 sample subjects over 14 years. In order to analyze the difference in dementia risk factors between men and women, three machine-learning-based dementia risk factor analysis models were constructed and compared. As a result of the experiment, it was found that the risk factors for dementia in men and women are different. In addition, not only did the results include most of the known dementia risk factors, previously unknown candidates for dementia risk factors were also identified. We hope that our research will be helpful in finding new dementia risk factors.

## 1. Introduction

Dementia makes elderly people unable to live their normal lives, while at the same time creating problems of social instability and financial burden [1]. As the world’s aged population grows, the prevalence of dementia is expected to increase. In 2015, there were about 47 million people with dementia worldwide (or about 5% of the world’s elderly population), and the number of people with dementia is expected to increase to 75 million in 2030 and 130 million in 2050 [1]. In Korea, there were 650,000 dementia patients among the elderly population aged 65 and over in 2016, but estimates suggest that the number of dementia patients will exceed 1 million in 2024 and 3 million in 2050 [2]. Drugs that are currently approved and marketed as dementia drugs delay the progression of dementia but do not fundamentally cure the condition [3]. As the center of dementia management shifts to prevention due to delays in drug development, the importance of identifying risk factors is gradually increasing. Currently, a lot of research on risk factors for dementia is being actively conducted, and the relation between dementia and various diseases is expected to steadily increase [3]. There are prominent factors associated with an increase in the prevalence of dementia, which is higher in women than in men [4]. As of 2018, the proportion of women in Korea who are aged 65 and over is 57.2% of which 62% have dementia [2]. Therefore, we investigated the related studies to examine the question of whether there are gender differences in dementia risk factors. Many studies have been conducted of gender differences in dementia risk factors [5,6,7], and several have been conducted using neuroimaging, such as Magnetic Resonance Imaging (MRI) and Positron Emission Tomography Scan (PET) [7,8].

Seo et al. [7] conducted a study of gender differences in dementia risk factors related to cardiac metabolic risk factors. MRI images were used to measure the relation between cortical thickness and cardiac metabolic risk, one of the dementia risk factors for both men and women. The decrease in cerebral cortical thickness (cortical atrophy) is a potential factor that can predict cognitive decline in normal patients as well as dementia patients, and studies show that the risk of Alzheimer’s dementia increases when the thickness of the cerebral cortex becomes too thin. A total of 1322 elderly people with normal cognitive function, including 774 men (58.5%) and 548 women (41.5%) over the age of 65, participated in a cross-sectional study using MRI. The results indicated the factors of cortical thickness reduction due to cardiovascular risk factors were high blood pressure, diabetes, and obesity in women and low weight in men. Elbejjani et al. [8] studied the effect of depression, a dementia risk factor, on dementia in men and women. Previous studies have indicated that hippocampal atrophy is related to dementia [8]. Over a four-year period, 1328 people aged 65–80 years participated in a study of the effect of depression on hippocampus size as measured by MRI. A multivariate linear regression model was used to analyze the results, which concluded that the relation between depression and a decrease in hippocampus size is correlated for women but not for men. In summary, depression is a dementia risk factor only for women. These two studies suggest that men and women have different dementia risk factors, and the same risk factors have differential effects on men and women. Since the two studies mentioned above were conducted directly on patients, the results are considered to be reliable. However, considerable time and funding are required to secure an appropriate number of subjects for statistical verification. In this paper, the gender differences in dementia risk factors were analyzed by using machine-learning techniques and the National Health Information System—Senior Cohort, which is a medical database containing data for hundreds of thousands of older people. The machine learning technique has the advantage of being able to detect a very small causal relation among data that is typically ignored by the existing statistical techniques [9]. The senior cohort used in our study is a research database built to support research on the elderly, including the identification of risk factors and prognosis analysis for diseases that affect seniors. Research using the senior cohort and machine learning has overcome the limitations of existing research methods and demonstrated high efficiency and effectiveness. First, the time–cost efficiency was improved by using the existing senior cohort. Second, the accuracy of the experimental results was improved because a large number of subjects were included in the analysis and machine learning was utilized. Lastly, since the model we proposed was built with all the data for the elderly, we presented and analyzed the known dementia risk factors as well as the unknown dementia risk factors for men and women. Because our approach uses cohort data, and access to research data is quick and easy, we believe that it will accelerate early dementia prediction research and contribute to discovering new risk factors for dementia.

## 2. Gender-Based Dementia Risk Factors Analysis

The design of this study was approved by the Institutional Review Board of Public, Korea National Institute for Bioethics Policy, Seoul, South Korea (P01-201611-21-002).

First, the gender differences in the dementia risk factors were analyzed using the senior cohort, which mainly included elderly patients with dementia. Second, in the feature selection process, step, the multi-layer perceptron (MLP) model, a machine-learning technique, was modified to be suitable for the study.

### 2.1. Introduction of Senior Cohort

**Figure 1:**
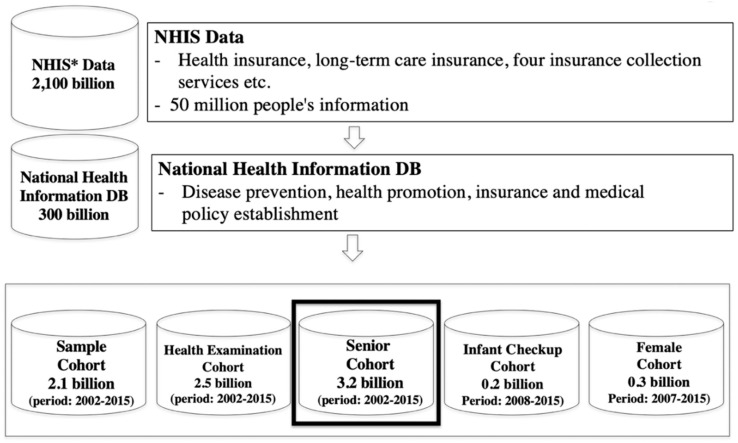
**NHIS** DB analysis.

The senior cohort used in this study is a highly reliable database provided by the National Health Insurance Corporation of Korea, which includes data for 97% (Korea National Health insurance subscriber) [10] of the national population [2]. The National Health Insurance Corporation (NHIC) of Korea was established in 1977 under the mission of improving the quality of people’s lives by promoting national health and social security. Since 1989, the NHIC has managed all medical expenses of medical recipients, medical providers, and government agencies in Korea. The NHIC operates the National Health Insurance Sharing Service (NHISS) to support policies and academic research using national health information data, and since 2002, the National Health Information Database (NHID), the world’s first national medical information database, has been made available to the public [2]. The NHIC has approximately 2100 billion records for 50 million people that it has accumulated during the course of the corporation’s work with health insurance, long-term care insurance, and four major insurance collection services. Of the entire database, 132 billion records were used to establish the NHID for producing information on the prevention of disease and promotion of health, establishment of health and medical policies, and improvement of medical quality. The NHID categories include the sample cohort, health screening cohort, senior cohort, infant and child screening cohort, and working women cohort (see Figure 1).

**Figure 2:**
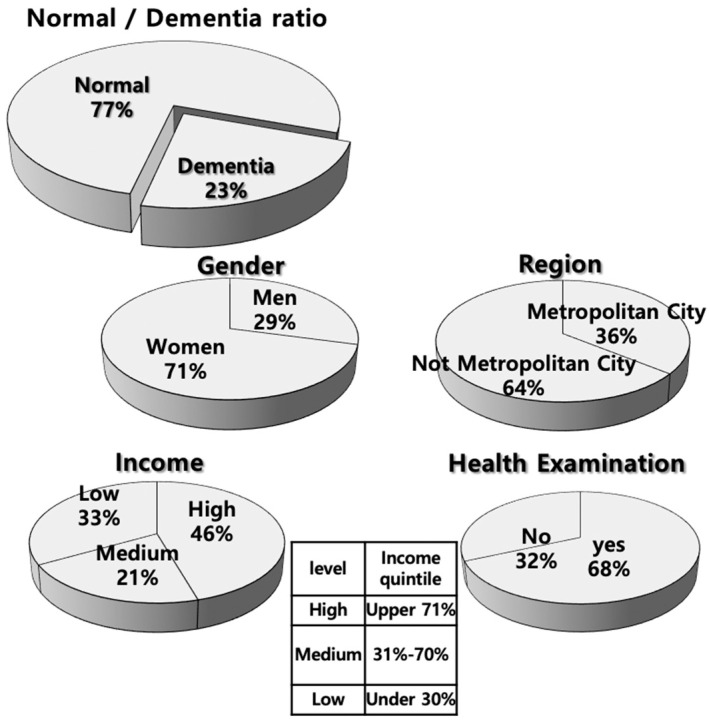
Distribution of senior cohort (as of 2013).

In this study, the senior cohort, which mainly includes data on elderly people with dementia, was used. The senior cohort consists of 558,147 elderly people and accounts for 10% of the total 5.5 million elderly population. The senior cohort is expected to be representative of the entire population of Korea. As of 2002, for the population aged 60 and over, data were generated from 2002 to 2013 (the latest data are from 2002 to 2015) to form a total of 672 levels by gender, region, and income level (see Figure 2). The main contents of the NHIS-senior cohort are Participant insurance eligibility DB (PIE-DB), Medical treatment DB (MT-DB), General health examination DB (GHE-DB), Medical care institution DB (MCI-DB), and Long-term care insurance DB (LCI-DB) (see Table 1).

### 2.2. Feature Selection

Personal medical information, such as demographic data, physical measurements, and medical records, is widely used to predict dementia and analyze risk factors. Feature selection was based on items from the PIE-DB, GHE-DB, and MT-DB of the NHIS-senior cohort (see Table 2). Items were not selected from the MCI-DB and LCI-DB, because they contain a limited number of elderly people related to long-term care, and the number of subjects was insufficient.

We grouped items from the PIE-DB, MT-DB, and GHE-DB in several ways. In PIE-DB, subjects’ gender is grouped into males and females, age has seven levels, and the income quintile has three levels. The disease information in MT-DB, which contains information on individual disease history, was used by mapping it with Korean Standard Classification of Diseases (KCD) version 7 [11]. KCD-7 codes are based on International Classification of Diseases of the World Health Organization (ICD) version 10 [12]. The structure of the original KCD-7 codes consists of seven digits. A total of 52,191 diseases are included in KCD-7 codes. In this study, to avoid data sparseness, 2087 groups of the first three digits were used as features.

In the GHE-DB, individual body information and medical examination information were grouped as normal abnormalities according to the “units for each major test item and the missing processing standard” (see Table 3). Since the senior cohort tracks patients for an extended period of time, we were able to utilize time-related changes such as increase/decrease and normal/abnormal personal medical information in each DB group as features. In addition, changes in an individual disease history over time was used as a feature.

When constructing the learning set, subjects were judged to have dementia if there was a record in the medical history information of MT-DB that indicated the individual was diagnosed with dementia. The KCD-7 codes of F00 (dementia in Alzheimer’s disease), F01 (vascular dementia), F03 (dementia of unknown detail), and G30 (Alzheimer’s disease) were considered dementia. Additional KCD-7 codes have been used to identify dementia, but these four KCD-7 codes or ICD-10 codes have been adopted in several previous studies [13,14].

One of the goals of this study is to find unknown risk factors for dementia. Therefore, “age”, the already specified risk factor for dementia, was excluded. This is because age can cause multicollinearity that can cause false positive. In order to solve the multicollinearity problem, in this study, the Pearson correlation coefficient was calculated for all the features, and features showing 0.7 or more were not used.

### 2.3. Risk Factors Analysis Model

Various machine-learning models were used to compare and analyze the data for men, women, and both men and women together to identify the optimal machine learning model. This study used the support vector machine (SVM), multi-layer perceptron (MLP), and convolutional neural networks (CNN), which showed excellent performance in various classification models in previous studies [15,16,17].

### 2.4. Support Vector Machine (SVM)

Support Vector Machine (SVM) [15], which is one of the supervised learning models, is applicable to nonlinear problems in addition to linear problems. The main purpose of SVM is to find the “optimal separation boundary hyperplane” that maximizes the margin between two classes. SVM transforms the data into a high dimensional space using kernel functions to linearly separate the two classes. The algorithm process of SVM is as follows: (1) select the optimal hyperplane to maximize the margin, (2) adjust the SVM loss function by adding a penalty for wrong classification, (3) in case the data are not linearly separated, it can be easily classified using the kernel trick that converts a linear model into a nonlinear model in a high-dimensional space. The ability to model nonlinear data is an advantage of SVM, as it operates smoothly in high-dimensional space. Additionally, since the degree of overfitting is less than that of a neural network technique, it is less likely for an error to occur during execution with training data and test data. The disadvantage of SVM is that as more samples are analyzed, the training time becomes longer, the memory allocation becomes larger, and performance worsens. In addition, it is necessary to adjust the kernel and model parameters several times to create an optimized model. SVM minimizes overfitting, as it searches for a decision surface to classify the data, and at the same time, learns by maximizing the distance between each dataset and the decision surface. Due to its advantages, SVM was considered the best algorithm in machine learning until deep-learning methods were developed.

### 2.5. Multi-Layer Perceptron (MLP)

Multi-layer perceptron (MLP) [16] is the most basic form of a feedforward neural network (FFNN). The feedforward neural network refers to a neural network in which only one direction of computation is defined from the input layer to the output layer. It is an artificial neural network structure that is widely used in pattern recognition problems. The multi-layer perceptron (MLP) is determined by calculating the weight and bias to minimize the error between the final output value and the actual value. The process of updating new weights and re-learning them with new weights to reduce errors in the feedforward algorithm is called a backpropagation algorithm. The advantage of MLP is its excellent generalization ability. Additionally, it can be applied to achieve various purposes, such as classification, prediction, evaluation, synthesis, and control. The disadvantage is that, as the build-up of a hidden layer increases and more perceptrons are created, the training time increases. In addition, there is a disadvantage of the backpropagation learning algorithm, which often fails to converge in the learning process and is prone to local minima, which can degrade performance. MLP is the most basic deep learning model. Deep-learning techniques have the advantage of learning patterns not able to be learned using existing machine-learning techniques. Therefore, there is a need for a formal study to compare MLP and SVM, which was considered the state-of-the-art technique before the development of deep-learning methods. In this study, to prevent overfitting, the number of data was increased, the dropout was set to 0.25, and L2 regularization was used.

### 2.6. Convolutional Neural Networks (CNN)

Convolutional neural networks (CNN) [17] are classified as deep neural networks in deep learning and mainly show good results for analyzing visual images. The basic concept of CNN is to make each element of the filter represented by a matrix automatically learn to be suitable for data processing. The area of acceptance in the convolutional layer is called a filter or kernel. This filter is the weight parameter of the convolution layer, which makes it suitable for finding the appropriate filter in the learning stage. CNNs learn directly from the data, use patterns to classify images, and eliminate the need to manually extract features. CNNs basically consists of a convolution layer and a pooling layer. The convolution layer applies weights and biases, and the pooling layer reduces the spatial size of the representation to reduce the number of network parameters or computational complexity. By sharing the weight of partial neural networks, the structure can be simplified further than the existing fully connected neural network method. Therefore, it is easy to learn a multi-layer structure through a backpropagation algorithm without prior learning. CNN is a method (algorithm) that optimizes the neural network, so that it can acquire a two-dimensional image by merging existing filter technology with the neural network. One advantage of CNN is that since all inputs have the same level of importance regardless of their location, CNN can successfully handle a fully-connected neural network even if the number of parameters greatly increases. In addition to images, CNN has shown good performance in identifying and classifying features of text, sound, and video. However, a disadvantage of CNN is that by itself it requires a vast amount of computation time, although this problem has been partially addressed by the increase in computing power and the arrival of the era of Graphics Processing Unit (GPU). The reason the CNN model was selected as a comparison model is because it was expected to produce different results than MLP, the most common deep-learning technique, by learning a different pattern and extracting various features from various locations. In this study, to prevent overfitting, the number of data was increased, the dropout was set to 0.25, and L2 regularization was used.

## 3. Experiment

**Figure 3:**
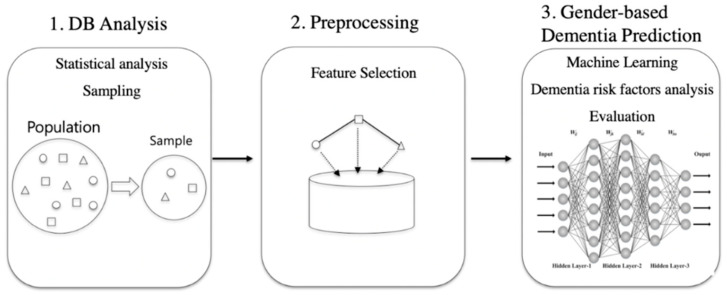
Workflow.

The experiment of this paper proceeded as shown in Figure 3. To confirm the difference in the dementia risk factors between men and women using the data from the senior cohort, dementia and normal groups were selected and tested for both men and women. The meaningful features were selected from the data and pre-processed, as described in Section 2.2, Feature Selection. Finally, the optimal model of machine learning was selected by comparing the various machine-learning models with dementia prediction models for men and women, and the dementia risk factors were extracted and analyzed.

### 3.1. Data Sampling

The sample consisted of data from 31,443 elderly people who had undergone a medical examination in 2013. Of the 31,443 elderly people, about 3000 elderly people who were diagnosed with dementia in 2013 and had medical examination records from 2011 to 2013 were sampled. The final sample for the experiment consisted on 3000 dementia and 3000 normal subjects who were randomly sampled.

Sampling was conducted for the integrated model of men and women and a model that separated men and women. First, for the male and female integrated model, the random sample included 3000 dementia and 3000 normal elderly subjects. For the gender separation model, the sample included 1500 men and 1500 women with dementia, and 1500 women of the 3000 normal elderly people.

### 3.2. Gender-Based Dementia Prediction Model

We compare the performance of MLP, SVM, and CNN models to select the optimal dementia prediction model for men and women. To learn the dementia risk factor analysis model, the backward greedy algorithm [18] was slightly modified. The backward greedy algorithm is a method for finding the optimal combination of qualities based on the weight of a feature by excluding one feature from the set with all features. In this study, the weight for each feature was calculated and analyzed by the method of the backward greedy algorithm.

There is a necessary observation period for dementia prediction and risk factor analysis. The optimal observation period for predicting dementia is discussed by Kim et al. [19]. Kim et al. researched the optimal observation period based on the integrated model, and the optimal period was 7 years (2007–2013). We also compared the optimal observation period. As a result of the experiment, the observation period was set to 3 years (2011–2013), because data over 3 years deteriorated the performance of predicting dementia. The model setup for the experiment is described in Table 4.

**Figure 4:**
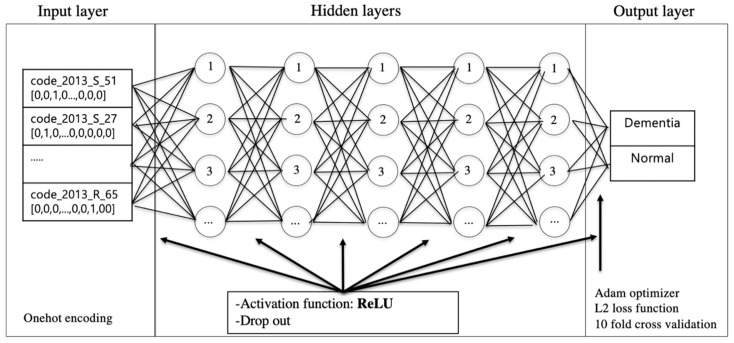
Multi-layer perceptron (MLP) structure.

The performance was evaluated by precision, recall, and F-measure scales through 10-fold cross-validation. The results of the experiments are shown in Table 5 for the performance of the MLP, CNN, and SVM models. The MLP model showed the best performance in both dementia prediction models for men and women. The structure of the MLP model is as shown in Figure 4.

## 4. Result

Risk factors were analyzed for each of the three dementia prediction models constructed in this experiment. Our experimental goal was to confirm that the risk factors for dementia in men and women are different through machine-learning experiments using the senior cohort rather than biodata, which are commonly used in medical research field. Therefore, a dementia risk factor analysis model was created for each gender and compared. In addition, to evaluate whether the risk factors for men and women with dementia were similar, an integrated model of men and women was created and compared with each model for the risk factors for dementia.

The left columns of Table 6 and Table 7, “Men’s RF Top 10” and “Women’s RF Top 10,” respectively, show the top 10 dementia risk factors obtained through the analysis model for men and women. The column on the right, “Men + Women RF Rank”, suggests how the ranking of the dementia risk factor changes when the information on the top 10 dementia risk factors for the other gender is added. In other words, it compares the ranking of risk factors for each gender to those for both gender to observe how it affects the prediction of dementia.

The first finding revealed by Table 6 and Table 7 is that men and women have different dementia risk factors. The second finding is that the risk factors for dementia in men and women should be analyzed separately. To confirm the fact that men and women should be analyzed separately, Table 6 and Table 7 illustrate that the ranking for most of the top 10 dementia risk factors of each gender were found to decrease when the genders were combined. In summary, the inclusion of both genders deteriorated the performance of dementia prediction for both men and women, indicating that the dementia risk factors should be analyzed separately by gender.

## 5. Discussion

Dementia risk factors for men and women verified in previous papers were also identified through the analysis of dementia risk factors based on our senior cohort study. Importantly, in addition to the previously known dementia risk factors, we also found additional candidates for dementia risk factors. In this paper, we analyzed the top 100 risk factors. Of these, 85 risk factors are the risk factors already verified by PubMed and Google Scholar, and the remaining 15 risk factor candidates are those that have not been suggested in dementia-related works. Table 8 and Table 9 show the dementia risk factor candidates. What can be contributed through our research results is that our study can promote the discovery of new risk factors and accelerate the development of early dementia prediction research. Meanwhile, the limitation of our study is multicollinearity that can cause false positive. To solve this problem, the Pearson correlation coefficient was calculated for all features, and features representing 0.7 or higher were not used.

## 6. Conclusions

Several conclusions were drawn from the results of predicting the gender-based dementia risk factors using cohort data. First, it was found that the risk factors for dementia in men and women are different. Second, the proposed model in this paper includes dementia risk factors that have been identified through previous research. Third, a candidate group for dementia risk factors was found, and further research is needed to verify them.

In this study, the analysis of dementia risk factors was focused on the elderly, which is the main age group that experiences dementia. However, dementia also occurs in the young. Therefore, we plan to use a sample cohort that includes not only the elderly but also the young. The list of the top 10 dementia risk factors presented in this paper is derived from the top 100 dementia risk factors identified in the current study. In the future, the analysis will be expanded from the current top 100 dementia risk factors to include additional risk factors. Additionally, the research may be conducted using other variables of interest such as differences in income and residential areas (city and countryside) that are beyond the research scope of this study, which was limited to the differences in dementia risk factors due to gender differences.

## Figures and Tables

**Table 1:** Senior cohort.

DB	Contents
Participant insurance eligibility DB (PIE-DB)	Demographics, socio-economic levels, and other data
Medical treatment DB (MT-DB)	Treatment items and treatment disease data
General health examination DB (GHE-DB)	Medical examination history from physical measurements to past medical records
Medical care institution DB (MCI-DB)	Data such as type of medical institution, area and installation period, number of hospital beds, number of doctors, and equipment availability status
Long-term care insurance DB (LCI-DB)	Long-term care application and decision results, opinions of doctors, such as an examination of recognized necessity, long-term care facility data

**Table 2:** Features.

DB	Features
Participant insurance eligibility DB (PIE-DB)	(1) Gender, (2) income quintile
Medical treatment DB (MT-DB)	Personal disease history diagnosis every year
General health examination DB (GHE-DB)	(1) Height, (2) weight, (3) body mass index, (4) waist, (5) blood pressure highest, (6) blood pressure lowest, (7) blood sugar before meals, (8) total cholesterol, (9) hemoglobin, (10) urine protein, (11) serum Glutamic Oxalacetate Transaminase (GOT), (12) serum Glutamic Pyruvate Transaminase (GPT), (13) gamma GTP, (14) history of personal illness: stroke, heart disease, high blood pressure, diabetes, hyperlipidemia, phthisis, cancer, (15) history of family illness: stroke, heart disease, high blood pressure, diabetes, cancer

**Table 3:** Normal/abnormal criteria of general health examination DB (GHE-DB) features.

No	Feature	Class	
		Normal	Abnormal
1	Body mass index (kg/m^2^)	0~29	30~300
2	Waist (cm)	Male: 50~90, Female: 50~85	Male: 90~130, Female: 85~130
3	Blood pressure highest (mmHg)	60~139	140~400
4	Blood pressure lowest (mmHg)	40~89	90~250
5	Blood sugar before meals (g/dL)	25~125	126~999
6	Total cholesterol (mg/dL)	40~229	230~999
7	Hemoglobin (g/dL)	Male: 12~16.5, Female: 10~15.5	Male: 0~12, Female: 0~10
8	Urine protein	Negative	Positive
9	Serum GOT (U/L)	0~50	51~999
10	Serum GPT (U/L)	0~45	46~999
11	Gamma GTP (U/L)	Male: 11~77, Female: 8~45	Male: 78~999, Female: 46~999

**Table 4:** Evaluation setting for each model.

Support Vector Machine (SVM)	Multi-Layer Perceptron (MLP)	Convolutional Neural Networks (CNN)
SVM type: C-SVC (classification)	Activation function: ReLU	Activation function: ReLU
Kernel type: radial basis function	Output layer: Sigmoid.	Output layer: Softmax
Loss: hinge loss	Dropout: 0.25	Dropout: 0.25
Epochs = 0.001	Optimizer: Adam	Optimizer: Adam
Batch size = 100	Loss: binary cross entropy	Loss: categorical cross entropy
Cache size = 40	Epochs = 15	Kernel size = 16
	Batch size = 1500	Batch size = 1500

**Table 5:** Model evaluation.

Models		Men	Women
Multi-Layer Perceptron (MLP)	Precision (%)	75.3	81.5
	Recall (%)	81.5	74.2
	F-score (%)	78.3	72.8
Support Vector Machine (SVM)	Precision (%)	81.7	70.7
	Recall (%)	66.4	58.6
	F-score (%)	73.3	65.3
Convolutional Neural Networks (CNN)	Precision (%)	68.6	55.8
	Recall (%)	62.4	75.3
	F-score (%)	65.4	64.1

**Table 6:** Comparison of top 10 dementia risk factors for men with risk factors for integrated sample.

Rank	Men’s Risk Factors Rank	Men + Women RF Rank
1	Other mental disorders due to brain damage and dysfunction and to physical disease	22
2	Paraplegia and tetraplegia	50
3	Vitamin D deficiency	73
4	Schizophrenia	10
5	Eating disorders	52
6	Other disorders of nervous system, NEC	32
7	Chronic kidney disease	98
8	Acute nephritic syndrome	94
9	Status epilepticus	51
10	Glomerular disorders in diseases classified elsewhere	84

Note: RF = Risk factor.

**Table 7:** Comparison of top 10 dementia risk factors for women with risk factors for integrated sample.

Rank	Women’s Risk Factors Rank	Men + Women RF Rank
1	Cerebral infarction	4
2	Other degenerative diseases of nervous system, NEC	8
3	Paraplegia and tetraplegia	50
4	Delirium, not induced by alcohol and other psychoactive substances	12
5	Inflammatory disease of uterus, except cervix	89
6	Unspecified urinary incontinence	92
7	Other disorders of pancreatic internal secretion	31
8	Other mental disorders due to brain damage and dysfunction and to physical disease	22
9	Vascular syndromes of brain in cerebro-vascular diseases	17
10	Depressive episode	2

Note: RF = Risk factor.

**Table 8:** Candidate group extracted from the top 100 men’s dementia risk factors.

Rank	Men’s Dementia Risk Factor Candidates
41	Diseases of thymus
42	Other disorders of adrenal gland
44	Other disorders of male genital organs
48	Hemiplegia
49	Somnolence, stupor, and coma
50	Urethral stricture

**Table 9:** Candidate group extracted from the top 100 women’s dementia risk factors.

Rank	Women’s Dementia Risk Factor Candidates
5	Inflammatory disease of uterus, except cervix
6	Unspecified urinary incontinence
12	Other disorders of adrenal gland
14	Enlarged lymph nodes
17	Polyp of female genital tract
28	Other symptoms and signs involving general sensations and perceptions
31	Hypofunction and other disorders of pituitary gland
44	Systemic inflammatory response syndrome
48	Diseases of thymus

## References

[1] World Health Organisation (2017). Global Action Plan on the Public Health Response to Dementia 2017–2025.

[2] Ministry of Health and Welfare (KR), National Institute of Dementia (KR) (2018). Korean Dementia Observatory 2018.

[3] Lao K., Ji N., Zhang X., Qiao W., Tang Z., Gou X. (2019). Drug development for Alzheimer’s disease: Review. J. Drug Target.

[4] Musicco M. (2009). Gender differences in the occurrence of Alzheimer’s disease. Funct. Neurol..

[5] Gannon O.J., Robison L.S., Custozzo A.J., Zuloaga K.L. (2019). Sex differences in risk factors for vascular contributions to cognitive impairment & dementia. Neurochem. Int..

[6] Mielke M.M., Vemuri P., Rocca W.A. (2014). Clinical epidemiology of Alzheimer’s disease: Assessing sex and gender differences. Clin. Epidemiol..

[7] Kim S.E., Lee J.S., Woo S., Kim S., Kim H.J., Park S., Lee B.I., Park J., Kim Y., Jang H. (2019). Sex-specific relationship of cardiometabolic syndrome with lower cortical thickness. Neurology.

[8] Elbejjani M., Fuhrer R., Abrahamowicz M., Mazoyer B., Crivello F., Tzourio C., Dufouil C. (2015). Depression, depressive symptoms, and rate of hippocampal atrophy in a longitudinal cohort of older men and women. Psychol. Med..

[9] Bzdok D., Altman N., Krzywinski M. (2018). Statistics versus machine learning. Nat. Methods.

[10] Comprehensive National Health Insurance Plan. http://medicare1.nhis.or.kr/hongbo/static/html/minisite/file/bojangnews82_1.pdf.

[11] Korean Standard Classification of Diseases. https://www.kcdcode.kr/browse/main/.

[12] International Classification of Diseases. https://www.who.int/classifications/icd/icdonlineversions/en/.

[13] Kang I.O., Lee S.Y., Kim S.Y., Park C.Y. (2007). Economic cost of dementia patients according to the limitation of the activities of daily living in Korea. Int. J. Geriatr. Psychiatry.

[14] Schwarzkopf L., Menn P., Leidl R., Wunder S., Mehlig H., Marx P., Graessel E., Holle R. (2012). Excess costs of dementia disorders and the role of age and gender-an analysis of German health and long-term care insurance claims data. BMC Health Serv. Res..

[15] Noble W. (2006). What is a support vector machine?. Nat. Biotechnol..

[16] Ruck D.W., Rogers S.K., Kabrisky M. (1990). Feature selection using a multilayer perceptron. J. Neural Netw. Comput..

[17] O’Shea K., Nash R. (2015). An introduction to convolutional neural networks. arXiv.

[18] Zhang T. (2011). Adaptive forward-backward greedy algorithm for learning sparse representations. IEEE Trans. Inf. Theory.

[19] Kim H., Chun H.W., Kim S., Coh B.Y., Kwon O.J., Moon Y.H. (2017). Longitudinal study-based dementia prediction for public health. Int. J. Environ. Res. Public Health.

